# Lumpfish (*Cyclopterus lumpus*) Is Susceptible to *Renibacterium salmoninarum* Infection and Induces Cell-Mediated Immunity in the Chronic Stage

**DOI:** 10.3389/fimmu.2021.733266

**Published:** 2021-11-22

**Authors:** Hajarooba Gnanagobal, Trung Cao, Ahmed Hossain, My Dang, Jennifer R. Hall, Surendra Kumar, Doan Van Cuong, Danny Boyce, Javier Santander

**Affiliations:** ^1^ Marine Microbial Pathogenesis and Vaccinology Laboratory, Department of Ocean Sciences, Memorial University of Newfoundland, St. John’s, NL, Canada; ^2^ Department of Bio-systems Technology, Faculty of Technology, University of Jaffna, Kilinochchi, Sri Lanka; ^3^ Aquatic Research Cluster, CREAIT Network, Ocean Sciences Centre, Memorial University of Newfoundland, St. John’s, NL, Canada; ^4^ Ocean Frontier Institute, Ocean Sciences Centre, Memorial University of Newfoundland, St. John’s, NL, Canada; ^5^ Southern Monitoring Center for Aquaculture Environment and Epidemic (MCE), Research Institute for Aquaculture No. 2, Ho Chi Minh City, Vietnam; ^6^ The Dr. Joe Brown Aquatic Research Building (JBARB), Ocean Sciences Centre, Memorial University of Newfoundland, St. John’s, NL, Canada

**Keywords:** Bacterial Kidney Disease (BKD), Gram-positive pathogen, *Renibacterium salmoninarum*, lumpfish, cell-mediated immunity

## Abstract

*Renibacterium salmoninarum* is a Gram-positive, intracellular pathogen that causes Bacterial Kidney Disease (BKD) in several fish species in freshwater and seawater. Lumpfish (*Cyclopterus lumpus*) is utilized as a cleaner fish to biocontrol sea lice infestation in Atlantic salmon (*Salmo salar*) farms. Atlantic salmon is susceptible to *R. salmoninarum*, and it can transfer the infection to other fish species. Although BKD outbreaks have not been reported in lumpfish, its susceptibility and immune response to *R. salmoninarum* is unknown. In this study, we evaluated the susceptibility and immune response of lumpfish to *R. salmoninarum* infection. Groups of lumpfish were intraperitoneally (i.p.) injected with either *R. salmoninarum* (1×10^7^, 1×10^8^, or 1×10^9^ cells dose^-1^) or PBS (control). *R. salmoninarum* infection kinetics and mortality were followed for 98 days post-infection (dpi). Transcript expression levels of 33 immune-relevant genes were measured in head kidney (*n* = 6) of fish infected with 1×10^9^ cells/dose and compared to the control at 28 and 98 dpi. Infected lumpfish displayed characteristic clinical signs of BKD. Lumpfish infected with high, medium, and low doses had a survival rate of 65%, 93%, and 95%, respectively. Mortality in the high-dose infected group stabilized after 50 dpi, but *R. salmoninarum* persisted in the fish tissues until 98 dpi. Cytokines (*il1β*, *il8a*, *il8b*), pattern recognition receptors (*tlr5a*), interferon-induced effectors (*rsad2, mxa*, *mxb*, *mxc*), and iron regulation (*hamp*) and acute phase reactant (*saa5*) related genes were up-regulated at 28 dpi. In contrast, cell-mediated adaptive immunity-related genes (*cd4a*, *cd4b*, *ly6g6f*, *cd8a*, *cd74*) were down-regulated at 28 dpi, revealing the immune suppressive nature of *R. salmoninarum*. However, significant upregulation of *cd74* at 98 dpi suggests induction of cell-mediated immune response. This study showed that *R. salmoninarum* infected lumpfish in a similar fashion to salmonid fish species and caused a chronic infection, enhancing cell-mediated adaptive immune response.

## Introduction

Bacterial Kidney Disease (BKD) caused by *Renibacterium salmoninarum* is a chronic disease of wild and cultured fish, including Atlantic salmon (*Salmo salar*), chinook salmon (*Oncorhynchus tshawytscha*), rainbow trout (*Oncorhynchus mykiss*), Arctic char (*Salvelinus alpinus* L.), Pacific herring (*Clupea pallasii pallasii*), sablefish (*Anoplopoma fimbria*), flathead minnow (*Pimephales promelas*), North Pacific hake (*Merluccius productus*), ayu (*Plecoglossus altivelis*), eel (*Anguilla anguilla*) and bivalve molluscs, in both fresh and marine waters (1–7). *R. salmoninarum* has primarily adapted to infect and persist in salmonids (8). However, *R. salmoninarum* experimentally infected and caused mortality in non-salmonids including, sablefish and Pacific herring, shiner perch (*Cymatogaster aggregate*), common shiner (*Notropis cornutus*), and flathead minnow, and it caused mortalities events in minnow (*Phoxinus phoxinus*) and three-spined stickleback (*Gasterosteus aculeatus*) (2, 9–12).

*R. salmoninarum* is a Gram-positive, slow-growing, fastidious, and facultative intracellular pathogen (7, 13), which persistence within wild and farmed fish populations is high (2). *R. salmoninarum* is the only marine bacterial pathogen that has been documented of both horizontal (i.e. from fish to fish) and vertical (i.e. from parent to progeny) transmission (2). *R. salmoninarum* has caused substantial losses in the salmonid aquaculture industry, affecting up to 80% and 40% of the Pacific and Atlantic salmon stocks, respectively (8). The poor efficacy of antibiotics and vaccines in BKD prophylaxis has stymied the control of this pathogen (14, 15).

Lumpfish (*Cyclopterus lumpus*), a globiform teleost native to the North Atlantic, is used as an eco-friendly cleaner fish to biocontrol sea lice (e.g., *Lepeophtheirus salmonis*) infestations in the Atlantic salmon aquaculture (16). Lumpfish reduces the utilization of chemotherapeutants against sea lice in Atlantic salmon farms, consequently its annual demands have significantly increased in the North Atlantic (17). Lumpfish health is critical for its optimal performance and elimination of potential risk of disease transmission between lumpfish and salmon (18, 19). *Pasteurella* sp., *Piscirickettsia salmonis*, *Vibrio anguillarum*, *Vibrio ordalii*, *Aeromonas salmonicida*, *Pseudomonas anguilliseptica*, *Moritella viscosa*, and *Tenacibaculum maritimum* have been reported to be primary bacterial pathogens in lumpfish (17). Although *R. salmoninarum* outbreaks have not been reported in lumpfish, due to the broad host range of *R. salmoninarum* (*i.e.*, salmonids, non-salmonids, bivalves and molluscs) and its horizontal transmission ability (20, 21), it is important to determine the susceptibility of lumpfish to *R. salmoninarum* and its potential risk for BKD. The risk of *R. salmoninarum* infection in lumpfish is significant because sea lice, like other blood-sucking ectoparasites, act as *R. salmoninarum* vector and could transfer *R. salmoninarum* from salmon to lumpfish and vice versa (22–24). *R. salmoninarum* transmission may occur as a result of the dynamic interplay between a susceptible host and virulent *R. salmoninarum* in an environmental context that facilitates such disease conditions (i.e., environmental stressors in the marine environment, high stocking densities in cultured conditions or parasitic infestations) (25, 26). For instance, horizontal transmission of *R. salmoninarum* between fish species like sockeye salmon (*Oncorhynchus nerka*) and chinook salmon (*O. tshawytscha*) has been reported (20, 21), and high biomass within sea cages and the free movement of seawater in and out of cages could increase the opportunity for disease transmission (27). Cleaner fish like lumpfish poses moderate risk of disease transmission to salmon (28). Transmission of amoebic parasite (*Paramoeba perurans*) from lumpfish to Atlantic salmon was demonstrated under controlled conditions (29). Though, the anticipated risk of infected lumpfish transmit bacterial disease to salmon is low, Atlantic salmon showed susceptibility to a lumpfish isolate of *M. viscosa* (28, 30). Thus, it could be possible that lumpfish act as an asymptomatic carrier and transmit disease threat to salmon (19). Several studies on the fish immune response to *R. salmoninarum* infection have been conducted in salmonids (31–34). However, the lumpfish susceptibility and immune response to *R. salmoninarum* infection is unknown. In addition, lumpfish is becoming an accessible model to study marine infectious diseases and teleost immunity (35).

Here, we evaluated the lumpfish susceptibility to a type strain of *R. salmoninarum* (ATCC 33209) and immune response at early and chronic infection stages. We determined that lumpfish is susceptible to *R. salmoninarum*, causing mortality and a chronic infection in the surviving individuals, similar to salmonid fish. The immune response profile of lumpfish head kidney at early and chronic infection stages showed that *R. salmoninarum* dysregulates the expression of transcripts with functional annotations related to pattern recognition, inflammation, cytokines, iron regulation, and cell-mediated adaptive immunity.

## Materials and Methods

### *Renibacterium salmoninarum* Culture Conditions and Inoculum Preparation

**Figure 1:**
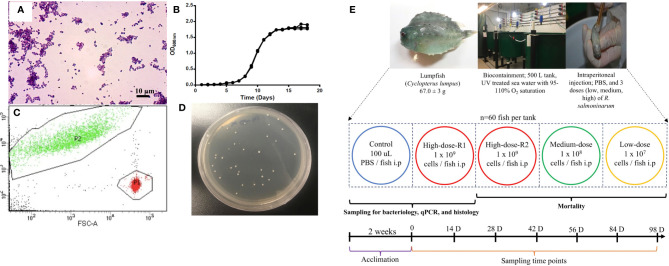
*Renibacterium salmoninarum* infection in lumpfish. **(A)** Characterization of *R. salmoninarum* by Gram staining; **(B)** *R. salmoninarum* growth curve in KDM2 broth; **(C)** Flow cytometric enumeration of *R. salmoninarum*. FSC: Forward Scatter; FITC: Green Fluorescence. In this plot of forward scatter *versus* fluorescence, green signals in the upper left-hand frame represent bacteria stained with the SYTO BC bacterial stain; red signals in the lower right-hand frame represent microsphere particles, which serve as the standard used to indicate sample volume. P1: Number of signals in the microsphere frame; P2: Number of signals in the bacterial frame. **(D)** *R. salmoninarum* colonies growing on a SKDM2 spread plate inoculated for quantitative culture of the bacterium from fish head kidney; **(E)** Experimental design for this study. 300 lumpfish (average weight: 67.0 ± 3 g) were divided in to 5 tanks (60 fish per tank) at the biocontainment facility. Fish from the control tank were intraperitoneally injected with 100 μl of PBS. Fish from the high (2 tanks for high dose; R1 and R2), medium, and low dose tanks were intraperitoneally injected with 100 μl of 10^9^, 10^8^, and 10^7^ cells per fish of *R. salmoninarum*, respectively. The mock-infected tank (PBS control) and 3 experimental tanks [low, medium, and high (R2 tank) doses of *R. salmoninarum*] were monitored for mortality. Fish for sampling of spleen, liver and head kidney were collected from the mock-infected tank and high dose R1 tank. Sampling time points were 0, 14, 28, 42, 56, 84, and 98 days post infection, and 6 fish were sampled at each time point.

*R. salmoninarum* type strain [ATCC (American Type Culture Collection) 33209] was cultured in complex KDM2 broth [1.0% (w/v) peptone (Difco), 0.05% (w/v) yeast (Difco), 0.05% (w/v) L-cysteine HCl (Sigma-Aldrich, St. Louis, MO, USA), 10% (v/v) fetal bovine serum (Gibco, Thermofisher, CA, USA), 1.5% (v/v) nurse medium contained filter-sterilized supernatant from *R. salmoninarum* cultures] (36) at 15°C with aeration in an orbital shaker (180 rpm). When required, KDM2 broth was supplemented with 1.8% (w/v) agar (Difco), and cycloheximide (0.005% (w/v); Sigma-Aldrich), D-cycloserine (0.00125% (w/v); Sigma-Aldrich), polymyxin-B sulfate [0.0025% (w/v); Sigma-Aldrich], and oxolinic acid [0.00025% (w/v); Sigma-Aldrich] to make *R. salmoninarum* selective KDM2 plates (SKDM2) (37). Bacterial growth was monitored by spectrophotometry (Genova Nano, Jenway, UK), flow cytometry (BD FACS Aria II flow cytometer and BD FACS Diva v7.0 software, BD Biosciences, San Jose, CA, USA) and/or by colony forming units (CFU) plate counting (38). The purity and integrity of bacterial cells were evaluated and confirmed by Gram-staining (39) (**Figure 1A**) and PCR (40, 41).

The bacterial infection inoculum was prepared as described previously (42), with modification for *R. salmoninarum*. Briefly, bacterial cells were cultured in 1 L of KDM2 at 15°C for 10 days and harvested at mid-logarithmic phase [Optical Density (O.D.) 600 nm = 0.8 ~1x10^8^ CFU mL^-1^] (**Figure 1B**) by centrifugation at 6,000 rpm for 10 min at 4°C, and washed once with sterile phosphate-buffered saline (PBS, pH 7.0; 136 mM NaCl, 2.7 mM KCl, 10.1 mM Na_2_HPO_4_, 1.5 mM KH_2_PO_4_) (43). The bacterial pellet was resuspended in 100 ml of PBS and subjected to bacterial enumeration using a bacteria counting kit (Invitrogen) and flow cytometry according to manufacturers’ instructions. The number of bacterial cells in the inoculum was calculated by dividing the number of signals in the bacterial frame by the number of signals in the microsphere frame (**Figure 1C**). The bacterial cells suspension was normalized to 3x10^10^ cells ml^-1^ and serially diluted in PBS to the final infection doses of 1×10^9^ cells dose^-1^ (high dose), 1×10^8^ cells dose^-1^ (medium dose), and 1×10^7^ cells dose^-1^ (low dose).

### Lumpfish

All animal protocols required for this research were reviewed and approved by the Institutional Animal Care Committee and the Biosafety Committee at Memorial University of Newfoundland (MUN) (https://www.mun.ca/research/about/acs/acc/) based on the guidelines of the Canadian Council on Animal Care (https://ccac.ca/). Experiments were conducted under protocols #18-01-JS, #18-03-JS, and biohazard license L-01.

Specific pathogen-free lumpfish (67.0 ± 3 g; mean ± SD) were produced and cultivated at the Joe Brown Aquatic Research Building (JBARB; Ocean Sciences Centre, St. John’s, NL, Canada). Infection studies were conducted in the aquatic level 3 (AQ3) biocontainment unit at the Cold-Ocean Deep-Sea Research Facility (CDRF; Ocean Sciences Centre, St. John’s, NL, Canada). Fish were distributed into five 500 L tanks (60 fish per tank) at a biomass of 25 kg m^-3^ and acclimated for 2 weeks at 10˚C before *R. salmoninarum* infection. Prior to and throughout the experimental study, fish were kept at optimal conditions (500 L tanks with flow-through (75 L min^-1^) filtered and UV-treated (8-10˚C) seawater, 95-110% air saturation, ambient photoperiod (12 h light:12 h dark). The fish were fed daily at a rate of 0.5% of their body weight per day with the commercial aquafeed Skretting - Europa 15 (55% crude protein, 15% crude lipid, and 1.5% crude fiber, 3% calcium, 2% phosphorus, 1% sodium, 5000 IU/kg vitamin A, 3000 IU kg^-1^ vitamin D, and 200 IU kg^-1^ vitamin E).

### *Renibacterium salmoninarum* Infection in Lumpfish

Lumpfish were intraperitoneally (i.p.) injected with 100 μl of 10^7^, 10^8^, or 10^9^ cells of *R. salmoninarum* dose^-1^, similar to infection studies in salmonids and other fish species (44–47). A duplicate group of lumpfish i.p. injected with the 10^9^ cells dose^-1^, was utilized for tissue sampling. Lumpfish i.p. injected with PBS were used as a control group (**Figure 1E**). Fish were monitored daily for mortality and clinical signs until 98 days post-injection (dpi) (**Figure 1E**). The survival rate was calculated according to Survival rate (%) = (Survivors at the end of the experiment/Initial individuals) × 100 (48).

Samples of spleen, liver, and head kidney were taken at 14, 28, 42, 56, 84, and 98 dpi from six lumpfish infected with 10^9^ cells of *R. salmoninarum* dose^-1^ and PBS injected lumpfish groups (**Figure 1E**). Before sampling, lumpfish were netted and euthanized with an overdose of MS222 (400 mg L^−1^; Syndel Laboratories, Vancouver, BC, Canada). Each tissue was aseptically collected and consistently subsampled for bacteriology, histology and immune-relevant transcript expression analyses. For bacteriology analysis, 30-100 mg of tissue was individually placed into a sterile homogenizer bag (Nasco whirl-pak^®^, USA), kept on ice and processed soon after harvesting (< 1 h). For histology, tissue sections were fully submerged into 15 ml falcon tubes containing 10% neutral-buffered formalin. For transcript expression analyses, 50-100 mg of tissue was placed in a 1.5 mL RNase-free tube, flash-frozen using liquid nitrogen, and stored at -80 ˚C until RNA preparation.

### Determination of Bacterial Load in Lumpfish Tissues

To study *R. salmoninarum* kinetics in lumpfish tissues, bacterial loads per g of tissue of infected lumpfish (*n* = 6, from high dose infected group) were determined at 14, 28, 42, 56, 84, and 98 dpi according to previously described procedures for *R. salmoninarum* isolation from salmonid kidney (49) with modifications. Briefly, tissues were kept cold on ice after extraction and during all the procedures. Tissue samples were aseptically weighed in the sterile homogenizer bag, suspended in PBS peptone [PBS (pH 7.4); 0.1% peptone] in the ratio of 1 mL PBS peptone per 0.1 g of tissue, and mechanically homogenized. Tissue homogenates were then transferred into sterile 1.5 mL centrifuge tubes and centrifuged at 2500 x *g* for 20 minutes at 4°C. The absence of bacteria in the supernatant was confirmed by sub-culturing 10 μL on SKDM2 plates. The pellet was resuspended in PBS peptone at a ratio of 1:1 (w/v) (i.e., 0.1 g of tissue was resuspended in 100 μl of PBS peptone) and mixed using Vortex mixer (Corning, Life Sciences, USA). The suspension was serially diluted in PBS peptone (1:10), and either 10 μl of the tissue homogenate or 10 μL of the serial 10-fold dilution was spread onto SKDM2 agar plates (**Figure 1D**). The plates were sealed with paraffin film to prevent desiccation and incubated at 15°C for up to 4-8 weeks. In each sampling point, the *R. salmoninarum* recovered on SKDM2 agar plates from lumpfish tissues were pure, and the observed *R. salmoninarum* colonies showed a homogenous morphology (**Figure 1D**). Also, the inocula obtained from these colonies were confirmed as *R. salmoninarum* by Gram-staining (i.e., presence of pure, Gram-positive diplobacilli) and PCR (i.e., positive amplification with the *R. salmoninarum* specific primers (40, 41). *R. salmoninarum* loads (CFU g of tissue^-1^) were quantified by dividing the number of colonies by the weight of tissue plated (i.e., for a starting tissue weight of 0.1 g, 10 μl of the homogenate was spread onto SKDM2, then the tissue plated was equivalent to 0.01 g).

### Histopathological Examination

Tissue samples of spleen, liver, head kidney collected at 14, 28, 42 and 98 dpi from PBS-control and high dose *R. salmoninarum* infected lumpfish groups were analyzed for histopathology. Tissues were fixed in 10% PBS-buffered formalin for three days at room temperature. The formalin was then removed and the fixed tissues were preserved in PBS at 4°C until processing for paraffin embedded tissue block according to established procedures (50). Tissue sections of 5 μm thickness were stained with hematoxylin and eosin (Leica Biosystems) using established protocols (51, 52) and observed for histopathological changes under the light microscope (Olympus CX40, USA).

### RNA Preparation

To study the lumpfish immune response to *R. salmoninarum* chronic infection, head kidney samples (*n* = 6 per group) extracted at 28 and 98 dpi from control (PBS-injected group) and infected lumpfish (10^9^ cell dose^-1^) groups were selected for real-time quantitative polymerase chain reaction (qPCR) analyses. Approximately 80-100 mg of tissue was added to a 1.5 mL RNase-free centrifuge tube containing 500 µL of TRIzol reagent (Invitrogen) and homogenized using a motorized RNase-Free Pellet Pestle Grinder (Fisherbrand, Fisher Scientific, USA). Then, additional 500 µL of TRIzol was added, mixed by pipetting, and RNA extractions were completed following manufacturer’s instructions. Extracted RNA samples were then purified using RNeasy MinElute Cleanup Kit (QIAGEN, Mississauga, ON, Canada) following manufacturer’s instructions. RNA samples were treated with TURBO DNA-free™ Kit (Invitrogen) for complete digestion of DNA and removal of remaining DNase and divalent cations, such as magnesium and calcium. Purified RNA samples were quantified and verified for purity using a Genova Nano microvolume spectrophotometer (Jenway, UK), and RNA integrity was tested by 1% agarose gel electrophoresis (43). All RNA samples used in this study showed acceptable purity ratios (A260/230 > 1.8 and A260/280 > 2.0) and integrity (28S and 18S ribosomal RNA bands at a 2:1 ratio) (**Supplementary Figure S1**).

### cDNA Synthesis and qPCR Parameters

First-strand cDNA templates for qPCR were synthesized in 20 μL reactions from 1 mg purified RNA using SuperScript IV VILO Master Mix (Invitrogen) following the manufacturer’s instructions.

PCR amplifications were performed in 13 ml reactions using 1X Power SYBR Green PCR Master Mix (Applied Biosystems), 50 nM of both the forward and reverse primers and the indicated cDNA quantity (see below). Amplifications were performed using the QuantStudio 6 Flex Real-Time PCR system (384-well format) (Applied Biosystems). The real-time analysis program consisted of 1 cycle of 50°C for 2 min, 1 cycle of 95°C for 10 min, 40 cycles of 95°C for 15 sec, and 60°C for 1 min, with fluorescence detection at the end of each 60°C step and was followed by dissociation curve analysis.

### Primer Design and Quality Assurance Testing

For each gene that was subjected to qPCR analyses, a group of transcripts (with associated TRINITY IDs) were obtained from the NCBI Sequence Read Archive (SRA) under accession number SRP238224 (**Supplementary File S1**). To confirm the identity of a given transcript, determine its orientation and identify the coding sequence (CDS), a BLASTx search of the non-redundant (nr) protein sequences database using a translated nucleotide query was performed between June and July 2019. A database of all confirmed transcript sequences for a given gene was created using Vector NTI (Vector NTI Advance 11.5.4, Life Technologies). Next, for a given gene, multiple sequence alignments were performed for its corresponding transcripts using AlignX (Vector NTI Advance 11.5.4). These alignments were used to determine if the transcripts were identical, contained single nucleotide polymorphisms (SNPs)/sequencing errors or represented different gene paralogues/isoforms. In the case of gene paralogues/isoforms, these alignments were also helpful to determine their percentage identity and to identify regions where paralogue/isoform-specific qPCR primers could be designed.

Primers were designed using Primer3 (53–55). However, in the case of the gene paralogues/isoforms, some were custom-designed in paralogue/isoform-specific areas to ensure specificity. All primers are located in the CDS and in an area, which overlapped with that of the best BLASTx-identified sequence. In the case of gene paralogues/isoforms, primers were designed in an area with ≥ 3 bp difference between them to ensure specificity. The amplicon size range was between 90-160 bp. The sequences, amplicon sizes and efficiencies for all primer pairs used in the qPCR analyses are presented in **Table 1**.

**Table 1:** qPCR primers used in this study.

Gene name (symbol)	Trinity ID (SRP238224)	Primer sequence (5′ to 3′)	R^2^	Amplification efficiency (%)	Amplicon size (bp)
**Genes of interest**					
*C-C motif chemokine-like 19* (*ccl19*)	DN10492_c0_g1_i4	F: GCTCAGGTACCAACGGACTG	0.999	88.4	94
		R: CGTGTCCTCCGATCTGTCTC			
*cyclooxygenase-2* (*cox2*)	DN750_c1_g1_i1	F: GAATTCCTCACCTGGGTCAA	0.994	90.6	122
		R: ATGGCATCTCTGAGGAAGGA			
*hepcidin anti-microbial peptide* (*hamp*)	DN2993_c0_g1_i4	F: GCTCGCCTTTATTTGCATTC	0.998	95.1	100
		R: ATATGCCGCAACTGGAGTGT			
*HLA class II histocompatibility antigen gamma chain* (*cd74*)	DN13708_c0_g1_i6	F: ACGCCAAGACACCTCTGACT	0.999	89.8	108
		R: GGAAGGTCTCGTTGAACTGC			
*immunoglobulin delta heavy chain* (*igh*δ)	DN1665_c0_g2_i7	F: GGAGACAGTGTTGTGCTGGA	0.999	88.4	121
		R: GGGCTTCAGGAAATTCAACA			
*immunoglobulin heavy chain variable region a* (*igha*)	DN1665_c0_g3_i2	F: AGGACTGGAGTGGATTGGAA	0.999	90.5	129
		R: TGCATGGTCTGTCCGTTTAG			
*immunoglobulin heavy chain b* (*ighb*)	DN1665_c0_g4_i1	F: GAATGGAACAAGGGGACAAA	0.999	89.6	108
		R: CGGTCGTTGAGTCTCTCCTC			
*immunoglobulin mu heavy chain a* (*ighma*)	DN121_c0_g3_i3	F: CAGCTTCTGGATTAGACTTTGA	0.998	90.2	107
		R: GATGTTGTTACTGTTGTGTTGG			
*immunoglobulin mu heavy chain b* (*ighmb*)	DN121_c0_g2_i2	F: CAGTCTCTAGGATATCATTCAG	0.992	92.1	101
		R: GTGGGTACCATCGTCACTATT			
*immunoglobulin mu heavy chain c* (*ighmc*)	DN121_c0_g3_i4	F: CAACATCCGGAATCACATTCAG	0.998	87.7	112
		R: GATTTTGAGGTCCCACTACCAT			
*interleukin 1 beta* (*il1β*)	DN22448_c0_g2_i1	F: ATTGTGTTCGAGCTCGGTTC	0.996	97.4	98
		R: CGAACTATGGTCCGCTTCTC			
*interleukin 8a* (*il8a*)	DN21169_c0_g1_i2	F: AAGTCATAGCCGGACTGTCG	0.999	96.3	109
		R: CCCTGCTGATGGAGTTGTCT			
*interleukin 8b* (*il8b*)	DN4613_c0_g1_i4	F: GTCTGAGAAGCCTGGGAGTG	0.996	87.3	138
		R: TCAGAGTGGCAATGATCTCG			
*interleukin 10* (*il10*)	DN41536_c0_g1_i1	F: AACCAGTGCTGTCGTTTCGT	0.986	97.8	106
		R: TGTCCAAGTCATCGTTTGCT			
*serum amyloid A* 5 (*saa5*)	DN111073_c0_g2_i2	F: AGAGTGGGTGCAGGAAAGAA	0.992	90.3	116
		R: GAAGTCCTGGTGGCCTGTAA			
*T-cell surface glycoprotein CD4a* (*cd4a*)	DN9678_c0_g2_i9	F: CGTTAAGGTGCTGCAGATCA	0.995	84.9	122
		R: GCGGAAACCATTTCAGTTGT			
*T-cell surface glycoprotein CD4b* (*cd4b*)	DN24146_c0_g1_i7	F: TGTGGGGTTAGCTCCTTCAC	0.996	94.2	138
		R: TGTTTGCGATCTCACCTTTG			
*lymphocyte antigen 6 complex locus protein G6f* (*ly6g6f*)	DN12606_c0_g1_i8	F: TCCATGTGGACGTGACTGTT	0.994	88.2	100
		R: AACGGTGTCTGAGCCTGAGT			
*T-cell surface glycoprotein CD8 alpha chain* (*cd8α*)	DN11791_c0_g1_i1	F: GCTTTGCTCTCTGGGCATAC	0.996	89.6	104
		R: TCCGGGTTCTTAAGTGGTTG			
*toll-like receptor 5a* (*tlr5a*)	DN29432_c0_g1_i1	F: TGGACGAGTTTCAGCAGTTG	0.988	95.6	129
		R: AGACCCCTCACATGTCCAAG			
*toll-like receptor 5b* (*tlr5b*)	DN55824_c0_g1_i5	F: CCATCATGCACTTTGTACGG	0.999	88.6	127
		R: TGCTGTTGATCTCCCTGATG			
*tumor necrosis factor alpha* (*tnfα*)	DN26791_c0_g1_i1	F: TTAGAAGGGAGCTGCGAAGA	0.982	90.1	119
		R: ATGACGATCCGGTTGTTCTC			
*ATP-dependent RNA helicase lgp2* (*lgp2*)	DN49186_c0_g1_i1	F: GCAACCTGGTGGTACGCTAT	0.998	84.9	104
		R: CTCGGCGACCACTGAATACT			
*C-C motif chemokine-like 20* (*ccl20*)	DN9266_c0_g1_i3	F: ATGGGCTACACCATCCAGAC	0.997	90.6	102
		R: CCACTTGGATGAAGGGTCAG			
*interferon gamma* (*ifnγ*)	DN81754_c0_g1_i1	F: CTCTGGCTGGTTGTCTGTCA	0.996	90.7	105
		R: TCGCTCTCTCGATGGAATCT			
*interferon regulatory factor 7* (*irf7*)	DN6933_c0_g1_i2	F: GGCTCATAGAGCAGGTGGAG	1.000	81.1	115
		R: CTGTCTTCGTCGTTGCAGTC			
*interferon-induced GTP-binding protein a* (*mxa*)	DN526_c0_g1_i6	F: TGCACAGACTCAAGCAGAGC	0.999	89.6	144
		R: CCACACTTGAGCTCCTCTCC			
*interferon-induced GTP-binding protein b* (*mxb*)	DN526_c0_g1_i3	F: TTGCGGCTTGGAAAAATATC	0.997	94.2	95
		R: TCCACGGTACCTTCGTTCAT			
*interferon-induced GTP-binding protein c* (*mxc*)	DN237_c1_g1_i1	F: GGAAGTGGCAGACATTGTGA	0.999	93.5	131
		R: CTGCTGCAATCTCCTTCTCC			
*radical S-adenosyl methionine domain containing protein 2*/*viperin* (*rsad2*)	DN16769_c0_g1_i1	F: AGGAGAGGGTGAAGGGAGAG	0.992	98.5	133
		R: ATCCAGAGGCAGGACAAATG			
*signal transducer and activator of transcription 1* (*stat1*)	DN3250_c2_g1_i2	F: CTCAAGATGCTGGACTGCAA	0.999	87.9	104
		R: ATGCTCTCGATCCACTTGCT			
*toll-like receptor 3* (*tlr3*)	DN30532_c0_g1_i1	F: AGAGGGCAGGGAATTTGAGT	0.999	92.9	101
		R: TGCACGAGTCATTCTCCAAG			
*toll-like receptor 7* (*tlr7*)	DN760_c1_g2_i1	F: GGCAAACTGGAAGAATTGGA	0.998	90.5	100
		R: GAAGGGATTTGAGGGAGGAG			
**Candidate normalizers**					
*60S ribosomal protein L32* (*rpl32*)	DN3569_c0_g1_i2	F: GTAAGCCCAGGGGTATCGAC	0.999	92.9	107
		R: GGGCAGCATGTACTTGGTCT			
*elongation factor 1 alpha* (*ef1α*)	DN12280_c0_g1_i3	F: CAAGGGATGGAAGATTGAGC	0.996	94.3	151
		R: TGTTCCGATACCTCCGATTT			
*eukaryotic translation initiation factor 3 subunit D* (*etif3d*)*	DN7623_c0_g1_i5	F: AGCCAGATCAACCTGAGCAT	1.000	90.3	134
		R: AGGCTGTACACCCGAATCAC			
*polyadenylate-binding protein 1 a* (*pabpc1a*)	DN6565_c0_g2_i3, DN6565_c0_g2_i4	F: CAAGAACTTTGGGGAGGACA	0.998	86.4	125
		R: TGACAAAGCCAAATCCCTTC			
*polyadenylate-binding protein 1 b* (*pabpc1b*)*	DN6565_c0_g2_i5	F: GACTCAGGAGGCAGCTGAAC	0.998	92.0	102
		R: TCGCGCTCTTTACGAGATTT			

Trinity IDs were associated with the groups of transcripts that were obtained from the NCBI Sequence Read Archive (SRA) under accession number SRP238224.

All Tm (melting temperatures) were set at 60°C by default during primer design using primer 3.

Amplification efficiencies were calculated using a 5-point 1:3 dilution series starting with cDNA representing 10 ng of input total RNA. See Materials and Methods for details.

*Expression levels of the transcripts of interest were normalized to expression levels of both etif3d and pabpc1b.

Each primer pair was quality tested to ensure that a single product was amplified (dissociation curve analysis) and that there was no primer-dimer present in the no-template control. Amplicons were electrophoretically separated on 2% agarose gels and compared with a 1 kb plus ladder (Invitrogen) to verify that the correct size fragment was being amplified (43). Amplification efficiencies (56) were calculated for both control and immune-stimulated cDNA pools from head kidney samples. Standard curves were generated for both cDNA pools using a 5-point 1:3 dilution series starting with cDNA representing 10 ng of input total RNA. The reported efficiencies are an average of the two values (**Table 1**).

### Endogenous Control (Normalizer) Selection

Expression levels of the genes of interest (GOIs) were normalized to expression levels of two endogenous gene controls. To select these endogenous controls, 5 genes [*60S ribosomal protein L32* (*rpl32*), *elongation factor 1-alpha* (*ef1a*), *eukaryotic translation initiation factor 3 subunit D* (*etif3d*), *polyadenylate-binding protein 1a* (*pabpc1a*) and *polyadenylate-binding protein 1b* (*pabpc1b*)] were analyzed. Briefly, the fluorescence threshold cycle (C_T_) values of all 24 samples in the study were measured (in duplicate) for each of these transcripts using cDNA representing 4 ng of input total RNA and then analyzed using geNorm (57). geNorm M values for all of the candidate normalizers were < 0.3, suggesting stable expression; however, *pabpc1b* (geNorm M = 0.165) and *etif3d* (geNorm M = 0.168) were selected as the two endogenous controls as they were the most stably expressed.

### Experimental qPCR Analyses

For experimental qPCR, head kidney samples from the control and from the high dose *R. salmoninarum* infected fish at both 28 and 98 dpi were chosen to represent early (28 dpi) and chronic (98 dpi) infection stages of *R. salmoninarum* based on the survival and head kidney colonization data (i.e*.*, fish showed mortality along with highest bacterial load at 28 dpi whereas fish mortality was stabilized even with the considerable amount of bacterial load at 98 dpi).

qPCR assays were designed for 33 transcripts with immune-relevant functional annotations (**Table 1**). These transcripts include pattern recognition receptors, cytokines, antimicrobial peptides, acute phase reactants, interferon regulators, interferon-induced effectors, humoral and cell-mediated adaptive immune response-related transcripts. An analysis of these transcripts-related innate and adaptive immunity would provide insight into host-pathogen interactions between lumpfish and *R. salmoninarum* at early and chronic infection stages.

The experimental qPCR analyses were conducted according to MIQE guidelines (58). cDNA representing 4 ng of input RNA was used as a template in the PCR reactions. All samples were analyzed on a single plate (3 GOIs and the two endogenous controls per plate; 33 GOIs over 11 plates). On each plate, for every sample, the GOIs and endogenous controls were tested in triplicate, and a no-template control was included. The relative quantity (RQ) of each transcript was determined using the QuantStudio Real-Time PCR Software (version 1.3) (Applied Biosystems) relative quantification study application, with normalization to both *pabpc1b* and *etif3d* transcript levels, and with amplification, efficiencies incorporated. For each GOI, the sample with the lowest normalized expression (mRNA) level was set as the calibrator sample (i.e., assigned an RQ value = 1) (**Supplementary Table S1**). Also, transcript expression levels were determined using the comparative 2^-ΔΔCt^ method (59–61) (**Supplementary Table S2**). The levels of transcript expression data from the 2^-ΔΔCt^ and the RQ data analysis methods were used in the main (**Figures 4**–**6**), and the supplementary (**Supplementary Figures S3** **–** **S5**) graphs, respectively.

### Statistical Analysis

All data are expressed as mean ± standard error (SE). Assumptions of normality and homoscedasticity were tested for the detected variances. Kaplan-Meier estimator was used to obtain survival fractions after the *R. salmoninarum* infection. The log‐rank test was used to compare the survival curve trends (*p*<0.0001), and a one-way ANOVA followed by Tukey’s multiple comparison *post hoc* test was used to determine significant differences between the survival of control and infected groups. Also, one-way ANOVA followed by the Holm-Sidak *post hoc* test was conducted to compare differences between tissues and within fish individuals at a single time point, whereas a non-parametric Kruskal-Wallis test was performed to compare the tissue bacterial loads between various time points per organ.

Transcript expression data were analyzed using a two-way ANOVA test, followed by the Sidak multiple comparisons *post hoc* test to identify significant differences between treatments (control and infected groups) at a single time point and for each treatment at different time points (i.e., 28 and 98 dpi). In all cases, *p* < 0.05 was considered statistically significant. All statistical analyses were performed using GraphPad Prism 8.0 (GraphPad Software, La Jolla California USA, www.graphpad.com).

## Results

### Lumpfish Survival, *R. salmoninarum* Infection Kinetics and Histopathology

**Figure 2:**
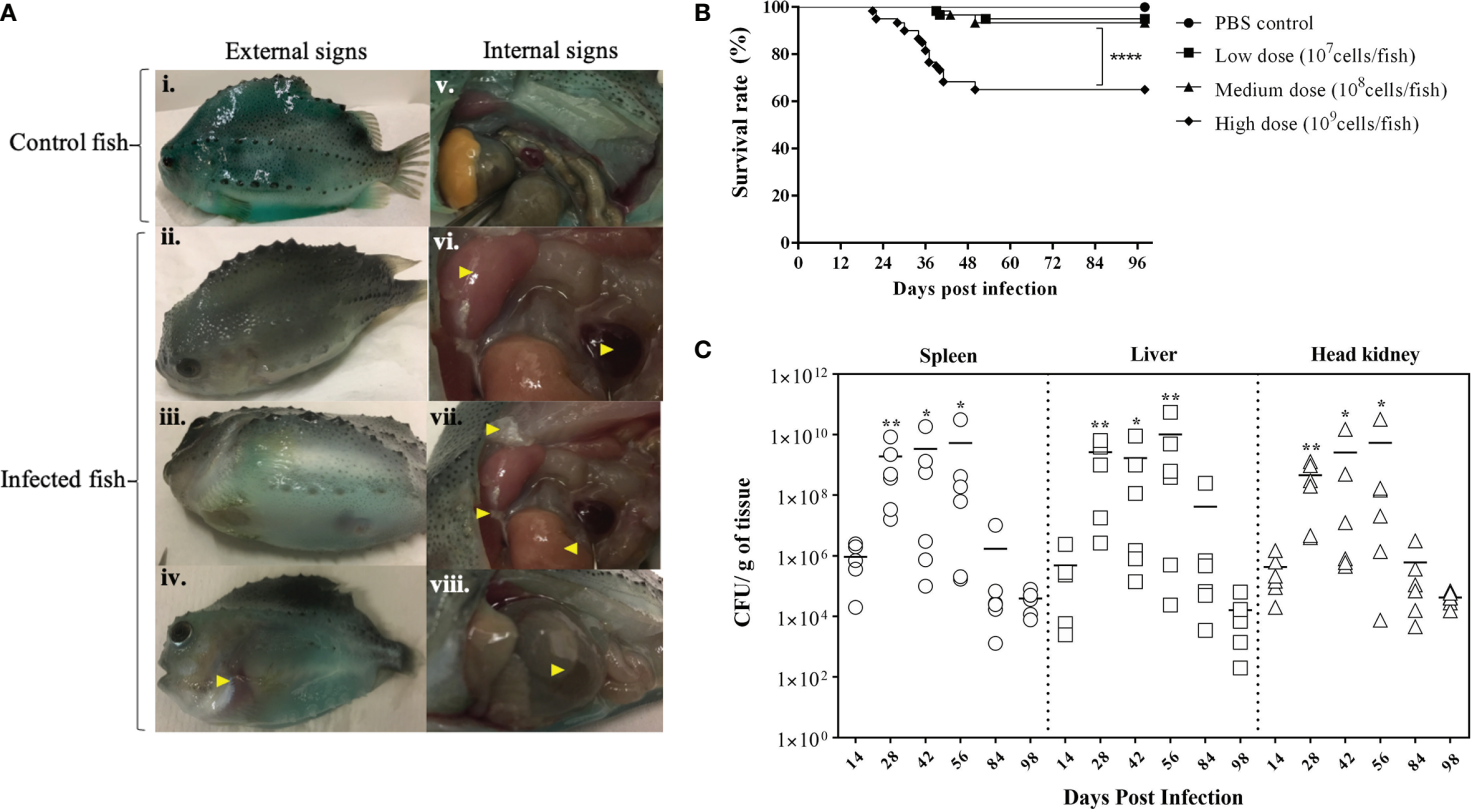
Bacterial Kidney Disease clinical signs, survival rates, and tissue colonization of *Renibacterium salmoninarum* infected lumpfish **(A)** Bacterial Kidney Disease signs and symptoms in lumpfish detected from 21 to 56 dpi. Pictures were randomly selected to visualize the external and internal signs compared to the control. Specific signs are indicated with yellow arrowheads. The external signs observed were (i) Control lumpfish; ii. Skin darkening; iii. Abdominal distension due to ascites; iv. Hemorrhages in ventral sites. The internal signs observed were v. Internal organs of control lumpfish; vi. Enlarged spleen and kidney; vii. Diffuse white membranous layer (pseudo membrane) on internal organs and pale liver; and viii. Accumulation of turbid fluid inside the abdominal sacs and cavities; **(B)** Percent survival of lumpfish exposed to experimental infection with high (1×10^9^ cells/fish), medium (1×10^8^ cells/fish) or low (1×10^7^ cells/fish) doses of *R. salmoninarum* compared to a PBS control; **** denotes the significant differences between infected and control groups (*p* < 0.001); **(C)** *Renibacterium salmoninarum* tissue colonization in lumpfish. Bacterial loads in spleen, liver and head kidney of lumpfish (*n* = 6) infected with the high dose (1 x 10^9^ cells/fish) of *R. salmoninarum* after 14, 28, 42, 56, 84, and 98 days post infection (dpi). Asterisks (*) represent significant differences (*p < 0.05, **p < 0.01) in the bacterial loads between time points (14, 28, 42, 56 and 84 dpi) per organ compared to the bacterial load at 98 dpi, as determined by the non-parametric Kruskal-Wallis test.

BKD is a slowly progressing systemic infection depending on the virulence of the *R. salmoninarum* strain that correlates with their number of major soluble antigen (*msa*) gene copies (2, 62). In this study, we used *R. salmoninarum* type strain ATCC 33209, which has only two *msa* copies (63), and it is known to exhibit lower pathogenicity, cause low mortality and a chronic infection in salmonids (36, 45, 64). Lumpfish infected with *R. salmoninarum* ATCC 33209 displayed characteristic clinical signs of a chronic BKD infection (**Figure 2A**). Mortality began at 20 dpi, gradually increased and stabilized after 50 dpi in the high dose infected group (1x10^9^ cells dose^-1^) (**Figure 2B**). In the medium (1x10^8^ cells dose^-1^) and low (1x10^7^ cells dose^-1^) dose groups, mortality began at 40 dpi and stabilized after 50 dpi as well (**Figure 2B**). External and internal BKD clinical signs and symptoms were observed in both dead and sampled fish. The clinical signs of *R. salmoninarum* infected lumpfish included hyper-pigmentation, lethargy, abdominal ascites, and hemorrhages in ventral sites. Examination of internal organs revealed splenomegaly, hydronephrosis, pale liver, pseudomembrane formation on internal organs, and ascites (**Figure 1F**). The survival rate for the high, medium, and low doses of *R. salmoninarum* groups was 65%, 93%, and 95%, respectively (**Figure 2B**). Cumulative number of fish mortalities (and mortality rate) observed during the experiment were 21 dead fish out of 60 total fish (35%), 4 dead out of 60 total fish (7%) and 3 dead out of 60 total fish (5%) for high, medium and low *R. salmoninarum* doses, respectively. The mortality data often considered the fish deaths from the tanks assigned for mortality observation (i.e., sampled fish were not considered in the analyses) (**Figure 1E**). Significantly lower survival (*p<*0.001) was observed in the high-dose *R. salmoninarum* infected group, whereas there were no significant differences in survival between PBS control, low and medium-dose fish groups.

*R. salmoninarum* colonized all of the organs sampled in the high-dose infected lumpfish (**Figure 2C**; **Supplementary Figure S2**). Significantly higher bacterial loads were observed at 28, 42, and 56 dpi compared to 98 dpi (**Figure 2C**). A substantial decrease in the bacterial load was observed at 84 and 98 dpi. Tissue colonization results correlated with the mortality data (**Figures 2B, C**).

**Figure 3:**
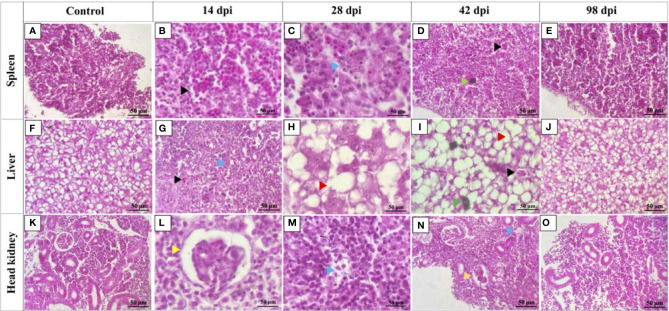
Histopathology changes in lumpfish tissues during *Renibacterium salmoninarum* infection. Lumpfish spleen, liver, and head kidney were collected from the high-dose (1 x 10^9^ cells/fish) infected group at 14, 28, 42 and 98 days post infection (dpi) and from the control (PBS-mock infected) group and stained with haematoxylin and eosin (H & E). Histopathological changes in lumpfish spleen: **(A)** Spleen section from control fish; **(B)** Spleen section from infected fish at 14 dpi, showing hemorrhages (black arrowhead); **(C)** Spleen section from infected fish at 28 dpi, showing degenerations (blue arrowhead); **(D)** Spleen section from infected fish at 42 dpi, showing hemorrhage (black arrowhead) and melanomacrophage center [MMC] (green arrowhead); **(E)** Spleen section from infected fish at 98 dpi. Histopathological changes in lumpfish liver: **(F)** Liver section from control fish; **(G)** Liver section from infected fish at 14 dpi, showing hemorrhage (black arrowhead) and degeneration (blue arrowhead); **(H)** Liver section from infected fish at 28 dpi, showing increased vacuolations (red arrowhead); **(I)** Liver section from infected fish at 42 dpi, showing hemorrhage (black arrowhead), MMC (Green arrowhead) and vacuolation (red arrowhead); **(J)** Liver section from infected fish at 98 dpi. Histopathological changes in lumpfish head kidney: **(K)** Head kidney section from control fish; **(L)**. Head kidney section from infected fish at 14 dpi, showing congested glomerulus (yellow arrowhead); **(M).** Head kidney section from infected fish at 28 dpi, showing degenerations (blue arrowhead); **(N).** Head kidney section from infected fish at 42 dpi, showing degeneration (blue arrowhead) and congested glomerulus (yellow arrowhead); **(O)**. Head kidney section from infected fish at 98 dpi. Stain: H & E; Magnification: ✕ 400.

In contrast to the control fish, spleen, liver, and head kidney of high dose infected fish at 14, 28, and 42 dpi showed apparent histopathological damages (**Figures 3B–D, G–I, L–N**). Tissue damage was observed in all three organs at 14, 28, and 42 dpi (**Figures 3C, G, M, N**). Hemorrhages were observed in the spleen and liver at 14 and 42 dpi (**Figures 3B, D, G, I**). The liver sections showed increased vacuolations in hepatocytes at 28 and 42 dpi (**Figures 3H, I**). Melanomacrophage centers were observed in the spleen and liver at 42 dpi (**Figures 3D, I**). Head kidney sections showed congested glomerulus with diffuse thickening of the basement membrane at 14 and 42 dpi (**Figures 3L, N**). Tissue sections of control fish and high dose infected fish at 98 dpi seemed similar without any significant histopathological damages (**Figures 3A, E, F, J, K, O**).

### Lumpfish Immune-Related Gene Expression in Response to *R. salmoninarum* Infection

The immune response of lumpfish to *R. salmoninarum* infection was evaluated in head kidney at 28 dpi and 98 dpi in 10^9^ cells dose^-1^ infected fish and compared to non-infected fish (PBS-control) at the same time points. Of the 33 genes (**Table 1**) that were evaluated, 12 genes were upregulated, and 4 genes were downregulated at both 28 and 98 dpi, whereas 17 genes were dissimilarly regulated.

**Figure 4:**
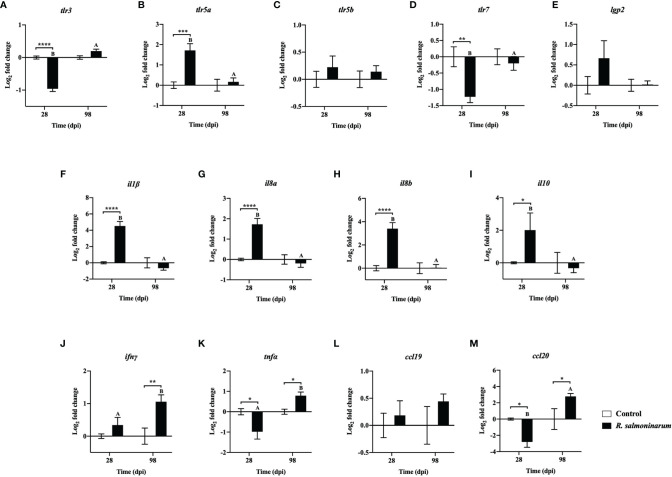
Expression of transcripts related to pattern recognition **(A-E)** and cytokines **(F-M)** in lumpfish head kidney in response to *R. salmoninarum* infection at 28 and 98 days post infection (dpi). Transcript expression levels in head kidney from control (PBS-mock infected group) and infected [high dose (1×10^9^ cells/dose) of *R. salmoninarum*] lumpfish at 28 and 98 dpi were analyzed using qPCR. Relative expression was calculated using the 2^−ΔΔCt^ method and log2 transformed; *etif3d* and *pabpc1b* were the endogenous control genes. A two-way ANOVA test, followed by the Sidak multiple comparisons *post hoc* test was used to identify significant differences between treatments (control and infected groups) at a single time point, and for a given treatment at different time points (28 and 98 dpi). Asterisks (*) represent significant differences between treatments at each time-point (*p < 0.05, **p < 0.01, ***p < 0.001, ****p < 0.0001). Different letters represent significant differences between control (lower case) and infected (upper case) groups at 28 compared to 98 dpi. Each value is the mean ± S.E.M (*n* = 6).

Thirteen genes related to pattern recognition (**Figures 4A–E**) and cytokines (**Figures  4F–M**) were differentially regulated. *toll-like receptor 3* (*tlr3*) and *toll-like receptor 7* (*tlrl7*) were significantly downregulated in infected fish compared to the control fish at 28 dpi (**Figures 4A, D**). *toll-like receptor 5a* (*tlr5a*) expression was significantly upregulated at 28 dpi compared to the respective control group (**Figure 4B**). *toll-like receptor 5b* (*tlr5b*) and *ATP-dependent RNA helicase lgp2* (*lgp2*) showed no significant differences in their expression levels between control and infected fish at 28 dpi and 98 dpi, respectively (**Figures 4C, E**).

Canonical proinflammatory cytokines encoding genes, including *interleukin 1 beta* (*il1β*), *interleukin 8a* (*il8a*), *interleukin 8b* (*il8b*), and the anti-inflammatory cytokine *interleukin 10* (*il10*), showed significantly higher expression in infected fish at 28 dpi compared to the non-infected control fish (**Figures 4F–I**). The expression levels of *il1β*, *il8a*, *il8b*, and *il10* in infected fish were not significantly different at 98 dpi compared to the control fish (**Figures 4F–I**).

*R. salmoninarum* infection significantly downregulated *tumor necrosis factor alpha* (*tnfα*) and *C-C motif chemokine-like 20* (*ccl20*) expression at 28 dpi (**Figures 4K, M**). In contrast, interferon *gamma* (*ifnγ*), *tnfα* and *ccl20* levels were significantly upregulated at 98 dpi compared to the respective non-infected fish group (**Figures 4J, K, M**).

**Figure 5:**
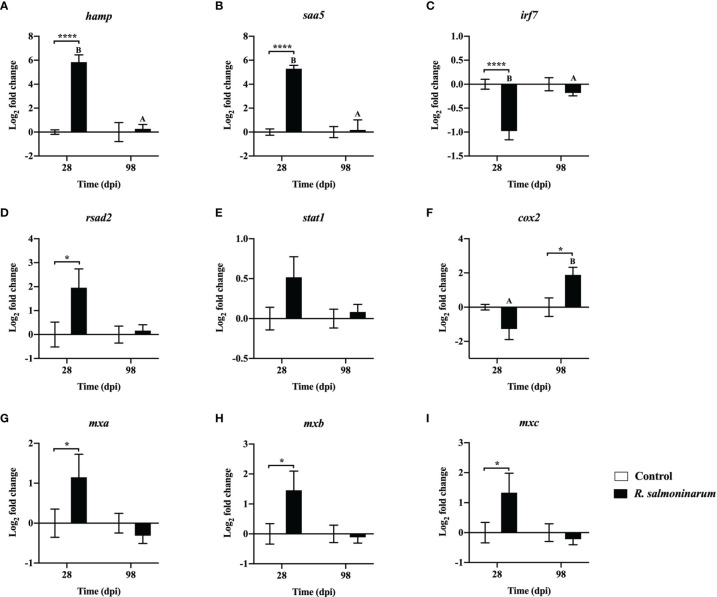
Expression of transcripts related to regulation of the innate **(A–E)** and inflammatory **(F–I)** immune response in lumpfish head kidney in response to *R. salmoninarum* infection at 28 and 98 days post infection (dpi). Transcript expression levels in head kidney from control (PBS-mock infected group) and infected [high dose (1×10^9^ cells/dose) of *R. salmoninarum*] lumpfish at 28 and 98 dpi were analyzed using qPCR. Relative expression was calculated using the 2^−ΔΔCt^ method and log2 transformed; *etif3d* and *pabpc1b* were the endogenous control genes. A two-way ANOVA test, followed by the Sidak multiple comparisons *post hoc* test was used to identify significant differences between treatments (control and infected groups) at a single time point, and for a given treatment at different time points (28 and 98 dpi). Asterisks (*) represent significant differences between treatments at each time-point (*p < 0.05, ****p < 0.0001). Different letters represent significant differences between control (lower case) and infected (upper case) groups at 28 compared to 98 dpi. Each value is the mean ± S.E.M (*n* = 6).

Expression levels of 9 genes regulating the innate (**Figures 5A–E**) and inflammatory (**Figures 5F–I**) immune response were assessed. Gene expression levels of *hepcidin antimicrobial peptide* (*hamp*) and *serum amyloid A 5* (*saa5)* were significantly upregulated in the head kidney of infected fish at 28 dpi compared to the respective non-infected control (**Figures 5A, B**).

At 28 dpi, *interferon regulatory factor 7* (*irf7*) was significantly downregulated (**Figure 5C**). Conversely, interferon-induced effectors such as *radical S-adenosyl methionine domain-containing protein 2*/*viperin* (*rsad2*) and three gene isoforms of *interferon-induced GTP-binding protein* (*mxa*, *mxb* and *mxc*) were significantly upregulated compared to the control fish at 28 dpi (**Figures 5D, G–I**). *cyclooxygenase-2* (*cox2*) expression was significantly upregulated in infected fish at 98 dpi compared to the control (**Figure 5F**).

**Figure 6:**
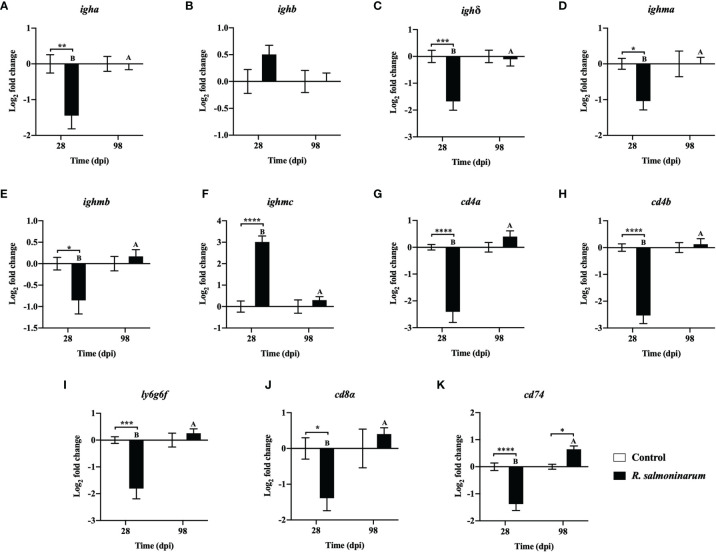
Expression of transcripts related to humoral **(A–F)** and cellular mediated **(G–K)** immunity in lumpfish head kidney in response to *R. salmoninarum* infection at 28 and 98 days post infection (dpi). Transcript expression levels in head kidney from control (PBS-mock infected group) and infected [high dose (1×10^9^ cells/dose) of *R. salmoninarum*] lumpfish at 28 and 98 dpi were analyzed using qPCR. Relative expression was calculated using the 2^−ΔΔCt^ method and log2 transformed; *etif3d* and *pabpc1b* were the endogenous control genes. A two-way ANOVA test, followed by the Sidak multiple comparisons *post hoc* test was used to identify significant differences between treatments (control and infected groups) at a single time point, and for a given treatment at different time points (28 and 98 dpi). Asterisks (*) represent significant differences between treatments at each time-point (*p < 0.05, **p < 0.01, ***p < 0.001, ****p < 0.0001). Different letters represent significant differences between control (lower case) and infected (upper case) groups at 28 compared to 98 dpi. Each value is the mean ± S.E.M (*n* = 6).

The expression levels of 11 genes playing putative roles in humoral (**Figures 6A–F**) and cellular-mediated adaptive immunity (**Figures 6G–K**) were assessed. Humoral (*immunoglobulin heavy chain variable region a* (*igha*), *immunoglobulin delta heavy chain* (*igh*δ), *immunoglobulin mu heavy chain a* (*ighma*), and *immunoglobulin mu heavy chain b* (*ighmb*)), and cellular-mediated (*T-cell surface glycoprotein CD4a* (*cd4a*), *T-cell surface glycoprotein CD4b* (*cd4b*), *lymphocyte antigen 6 complex locus protein G6f* (*ly6g6f*), *T-cell surface glycoprotein CD8 alpha chain* (*cd8α*), and *HLA class II histocompatibility antigen gamma chain* (*cd74*)) adaptive immunity-related genes showed significant downregulation at 28 dpi in the infected head kidney compared to the non-infected control (**Figures 6A, C–E, G–K**).

At 98 dpi, only one adaptive immune-related gene, *cd74*, was significantly upregulated in infected fish compared to the control (**Figure 6K**). Expression of most of the genes related to humoral and cellular-mediated immunity in infected fish at 98 dpi was restored to similar levels observed in the control fish (**Figures 6B, D, E–K**).

The qPCR results were similar between the 2^-ΔΔCt^ and the RQ data analysis methods. However, a few differences in the significance levels were detected for *tlr3*, *tnfα*, *rsad2*, *ighma* and *cd8α* expression at 28 or 98 dpi (**Figures 4A, K**; **5E**; **6D, J** and **Supplementary Figures S3A, K**; **S4E**; **S5D, J**).

## Discussion

As previously mentioned, lumpfish are in close contact with salmon when delousing sea lice in sea cage aquaculture (17, 19), and this interaction could result in the horizontal transmission of infectious disease agents between both species, including *R. salmoninarum*. Atlantic salmon is susceptible to *R. salmoninarum*, and it could transfer this pathogen to other fish species (20, 21). It is believed that lumpfish could act as a non-symptomatic carrier and transmit disease to cohabitating salmon (19). Haugland et al. (29) confirmed the experimental transmission of amoebic parasite from lumpfish to salmon (29). Also, Atlantic salmon susceptibility to a lumpfish isolate of *M. viscosa* reflecting the disease risk to salmon (30). Although BKD episodes have not been reported in lumpfish, its susceptibility and immune response to *R. salmoninarum* are unknown. Here, we examined the susceptibility of lumpfish to *R. salmoninarum* (ATCC 33209) type strain, which has been utilized for several infection studies in different fish species. Using similar *R. salmoninarum* infection doses like other studies, we also determined the infection kinetics and lumpfish molecular immune response at early and late chronic infection with *R. salmoninarum*. This study is the first report of *R. salmoninarum* experimental infection on lumpfish and provides immune-relevant information on how the lumpfish respond to *R. salmoninarum*.

For the *R. salmoninarum* infection kinetics studies, we selected the plate counting method in SKDM2 over typical methods for *R. salmoninarum* quantification (e.g., FAT, ELISA, and PCR) because it directly enumerates viable bacteria (7). Spleen and liver were also analyzed, in addition to the head kidney, to consider non-kidney *R. salmoninarum* infections, which have been well described in salmonids (65, 66). In lumpfish, *R. salmoninarum* infection becomes evident at 2 weeks post-infection, similar to chinook salmon (*O. tshawytscha*) i.p. infected with 1×10^6^ *R. salmoninarum* cells dose^-1^ (67). In an antibody capture ELISA and western blot based analysis, Turaga *et al.* (1987) reported that levels of *R. salmoninarum* soluble antigens in infected coho salmon (*Oncorhynchus kisutch*) gradually increased during the course of infection, and peaked at 20 dpi and thereafter, fish mortality was observed (68). Although in the current study we did not measure *R. salmoninarum* soluble antigen levels, mortality of *R. salmoninarum* infected lumpfish started at 20 dpi, similar to coho salmon, i.p. infected with *R. salmoninarum* cells in the exponential phase of growth (O.D. 500 nm = 1.0) (68). This suggests that mortality could be initiated by the accumulation of *R. salmoninarum* MSA in the infected lumpfish. Because increased MSA levels correlated with the severity of infection and mortality (68, 69).

Lumpfish infected with a lethal dose of *R. salmoninarum* showed prominent BKD associated clinical signs at 14, 28, and 56 dpi (**Figure 2A**), similar to clinical signs described in other fish species (7). Bacterial loads in spleen, liver and head kidney at various time points indicated that *R. salmoninarum* established an infection in all infected individuals (**Figure 2C**). In contrast, carp (*Cyprinus carpio* L.), a non-salmonid-like lumpfish, showed resistance to *R. salmoninarum* infection (4.8×10^7^ and 4.8×10^8^ cells/dose), and no bacteria were recovered from head kidney after infection (47). These results indicate that the lumpfish is susceptible to *R. salmoninarum* and could be a potential vector for this pathogen.

Significantly higher tissue bacterial loads at 28, 42, and 56 dpi correlated with higher mortality (**Figures 2B, C**). However, after fish mortality ended (**Figure 2B**), *R. salmoninarum* remained in the internal tissues (**Figure 2C** and **Supplementary Figures S2E, F**), indicating a pattern of chronic infection. Arctic charr (15), chinook salmon (62), lamprey (70) and carp (47) cleared *R. salmoninarum* infection after 175, 115, 92 and 38 days, respectively. *R. salmoninarum* persisted in lumpfish tissues at least for 98 dpi, which is consistent with studies in chinook salmon where *R. salmoninarum* caused a chronic infection and persisted for up to 100 dpi (36). However, if the current study had been extended, it is possible that lumpfish could have cleared the *R. salmoninarum* after 98-100 dpi, as seen in the Arctic charr (15) or remains in other tissues like gonads (i.e., *R. salmoninarum* in ovarian fluid is an important source of infection for the eggs) to facilitate vertical transmission (7).

The lethal dose 50 (LD_50_) of *R. salmoninarum* ATCC 33209 in various salmonid hosts ranged from 1.4×10^5^ to 2.94×10^8^ CFU dose^-1^ (64). We could not determine the LD_50_ for *R. salmoninarum* ATCC 33209 in lumpfish because the fish infected with the highest dose (1×10^9^ cells dose^-1^), similar to other studies, showed only 35% mortality. In contrast, mortality reached 100% within 15 days in Atlantic salmon infected with 10^8^ cells dose^-1^ of highly virulent *R. salmoninarum* strains (71). The LD_50_ of *R. salmoninarum* type strain in lumpfish might be greater than 1×10^9^ CFU dose^-1^, and although 10^9^ cells dose^-1^ of *R. salmoninarum* ATCC 33209 was sufficient to invade, replicate, and establish an infection in lumpfish, its lethality was lower than in salmonid species (36, 72–75).

Differences in virulence between *R. salmoninarum* isolates from several geographical regions and fish hosts have been reported (64). Rhodes et al. (76) demonstrated the positive correlation between the functional *msa* gene copy number per bacterial cell and virulence (i.e., increased mortality) (76). The type strain *R. salmoninarum* ATCC 33209 used in this study has two *msa* gene copies, and both of these *msa* gene copies are essential for disease development and mortality (62, 63). Compared to other *R. salmoninarum* strains, *R. salmoninarum* ATCC 33209 has a reduced virulence. For example, this strain showed lower virulence in chinook and coho salmon compared to the other isolates, and it is not capable of causing BKD in rainbow trout (45, 64). Furthermore, *R. salmoninarum* type strain does not infect the carp (*Epithelioma papillosum*) cell line, even with a dose of 1×10^9^ cells, in contrast to more virulent strains of *R. salmoninarum* (e.g., FT10) that are capable of invading and proliferate in these cells (45, 77). *R. salmoninarum* ATCC 33209 type strain was isolated in 1974, and it has been subjected to extensive laboratory passages, which may have contributed to its relatively reduced virulence (76). In the present study*, R. salmoninarum* ATCC 33209 was unable to kill all infected lumpfish even at a high dose, and this could be linked to the low virulence documented for *R. salmoninarum* ATCC 33209.

Histology observations in the sampled lumpfish infected with *R. salmoninarum* showed similarities with the histopathological characteristics of BKD in salmonids (**Figure 3**). For instance, glomerulopathy is related to antigen-antibody complexes deposition in the glomeruli, which causes thickening of the glomerular basement membrane (10, 78). In concordance with BKD histopathology, congested glomeruli were observed in the head kidney of infected lumpfish at 14 and 42 dpi (**Figures 3L, N**). Also, lysed and disrupted melanomacrophages resulting from the dispersal of pigments in tissues during BKD (79, 80) were observed in spleen and liver from infected lumpfish at 42 dpi (**Figures 3D, I**). No histopathological differences were observed at 98 dpi (**Figure 3**). The persistence of *R. salmoninarum* in lumpfish tissues at 98 dpi was indicative of a chronic infection, and the bacterium may remain dormant or controlled by the fish immune system (81). The lack of tissue inflammation and damage at 98 dpi could be explained by the known immune-suppressive nature of *R. salmoninarum* (8, 10, 82).

At 98 dpi, *R. salmoninarum* was isolated from spleen, liver and headkidney of the high dose infected lumpfish which showed no external, internal and histopathological disease signs (**Figures 2C** and **3**). Similar to our results, *M. viscosa* was isolated from kidneys of non-symptomatic lumpfish at 27 days post bath challenge (30). This implies that lumpfish could be asymptomatic carriers for *R. salmoninarum*, and chronic infection could be a common strategy of marine bacterial pathogens.

The BKD-related histopathology observations in lumpfish coincided with the downregulation of immune-related genes in lumpfish head kidney after *R. salmoninarum* infection. For instance, we observed that *R. salmoninarum* influenced the expression of genes related to pathogen recognition, immune signalling, antibacterial activity, and humoral and cell-mediated immunity in lumpfish (**Figures 4**–**6**).

TLR5 is associated with flagellin detection (83). *tlr5a* was significantly upregulated at 28 dpi (**Figure 4B**). Increased expression of *tlr5a* in lumpfish upon exposure to Gram-positive, non-motile or non-flagellated bacteria like *R. salmoninarum* (84) is controversial. However, a similar upregulation of *tlr5* in response to alive and formalin-killed *R. salmoninarum* has been reported (46, 85). Also, increased expression of *tlr5a* and *tlr5b* was reported in turbot (*Scophthalmus maximus* L.) mucosal tissues (i.e., intestine and gills) in response to the Gram-positive non-flagellated pathogen *Streptococcus iniae* (86). Therefore, the role of TLR5 beyond the recognition of flagellin, specifically in infection with non-flagellated bacteria in teleosts, warrants further investigation.

*R. salmoninarum* increased gene expression levels of the proinflammatory cytokine (*il1β*) and of the proinflammatory response related chemokines (*il8a*, *il8b*) at 28 dpi (**Figures 4F–H**) in lumpfish, which coincided with a canonical innate immune response. Simultaneously, *il10*, an anti-inflammatory mediator, was significantly upregulated at 28 dpi (**Figure 4I**). This pattern strongly suggests an *R. salmoninarum* induced immune suppression (8, 82). Similar to our results, IL-10 induction upon *R. salmoninarum* strain H-2 infection in Atlantic Salmon Kidney (ASK) cell line was observed by Bethke et al. (87) (87). IL10 counteracted the induced inflammatory immune responses (e.g., ILβ, IL8), and as a result, the pathogen could move forward in disease progression. However, as teleost fish IL-10 demonstrates immune suppressive function, *il-10* expression upon pathogen infection could be the natural way of lumpfish to regulate its early innate immune responses (88). Thus, IL-10 upregulation might be seen from a host point of view in which host is trying to create a conducive environment to alleviate host-mediated pathology. For instance, IL-10 can promote tissue repair to overcome the tissue damage due to disease progression (89).

IL-1β was activated in fish leucocytes and macrophages and induced the expression of proinflammatory transcripts such as *cox2* and *tnfα* (90–92). However, at 28 dpi, we observed that *cox2* and *tnfα* were not upregulated even with high expression of *il1β* (**Figures 4F, K**, and **5C**). IL-1β can also initiate an acute phase response and induce the synthesis of acute-phase proteins (APPs) such as serum amyloid A5 (SAA5) upon invasion of the pathogen (93, 94). We observed a significant upregulation of *saa5* at 28 dpi in lumpfish (**Figure 5B**) indicating an inflammatory response to the infection (95).

TNF-α is associated with inflammation and chronic infections (96). TNF-α can either improve the phagocytic activity of fish leucocytes or support the intracellular survival of pathogens (97–100). In the current study, despite the high bacterial load in the fish tissues (**Figure 2C**), significant downregulation of *tnfα* at 28 dpi was observed in lumpfish (**Figure 4K**), which could affect the *tnfα* dependent killing pathways, thereby facilitating the infection and intracellular survival of *R. salmoninarum* (32). Also, this *tnfα* repression could reflect the immune-suppressive action of *R. salmoninarum* in lumpfish.

Reducing the availability of iron to bacteria as a means of nutritional immunity is one strategy used by vertebrates such as fish to control pathogens (101). On the other hand, intracellular bacteria compete for iron for their survival (102). HAMP is an antimicrobial peptide (AMP) that has anti-bacterial and immuno-modulatory functions and plays a role in iron homeostasis in fish (103). Here, we found that *hamp* was significantly upregulated at 28 dpi (**Figure 5A**). Similar to our results, increased expression of *hamp* in head kidney of Atlantic salmon has also been observed with *R. salmoninarum* infection (46, 85). Additionally, *transferrin*, an AMP encoding gene, which has a putative role in iron sequestration from bacteria, is upregulated in response to *R. salmoninarum* in salmonid hosts (67) and is involved in BKD resistance in coho salmon (*O. kisutch*) (104). Thus, *hamp* and *transferrin* in lumpfish might play an essential role in the BKD response.

IFN-γ is associated with adaptive immunity and has a role in both the early and late immune responses and in the host immune defense to intracellular bacteria (96, 102). *ifnγ* stimulation in lumpfish at 28 dpi was not significant in our study. In contrast, significant upregulation of *ifnγ* was reported in Atlantic salmon and chinook salmon infected with *R. salmoninarum* (46, 67). Interferon-induced effectors (*rsad2, mxa*, *mxb* and *mxc*) were significantly upregulated at 28 dpi (**Figures 4J** and **5D, G–I**). Similar to our results, upregulation of *rsad2* has also been observed in Atlantic salmon head kidney upon *R. salmoninarum* infection (46). In addition, increased expression of mx genes, *mx1*, *mx2*, and *mx3* in rainbow trout macrophages (32) and *mx1* in chinook salmon (67), after *R. salmoninarum* infection was also reported.

The immune-suppressive effects of *R. salmoninarum* were also observed in the adaptive immune response of lumpfish at 28 dpi. For instance, significant downregulation of humoral (*igha, igh*δ, *ighma*, *ighmb*) (**Figures 6A, C–E**) and cell-mediated (*cd4a*, *cd4b*, *ly6g6f*, *cd8α*, *cd74*) (**Figures 6G–K**) adaptive immune-related transcripts at 28 dpi, was observed. Mortality in lumpfish during the early time points could be attributed to this immune suppressive function of *R. salmoninarum* observed at 28 dpi. Significant downregulation of *cd74* (an invariant polypeptide involved in major histocompatibility complex-II (MHC-II) formation and transport) (**Figure 6K**) in lumpfish head kidney at 28 dpi suggests that the T-cell responses could be modified towards an enhanced MHC-I and a reduced MHC-II dependent pathway, perhaps caused by an increased amount of MSA, similar to *R. salmoninarum* infection in rainbow trout (32, 105). This skewing towards the MHC-I pathway in lumpfish at the early stages of *R. salmoninarum* infection correlates with the BKD-dependent *major histocompatibility-1* (*mh1*) induction observed in Atlantic salmon at 13 dpi (46). Further, Rozas-Serri et al. (106) demonstrated that the humoral and cell-mediated adaptive immune responses against *R. salmoninarum* in Atlantic salmon pre-smolts were significantly downregulated at the later stage of infection (55 dpi) (106), which agrees with our findings at 28 dpi. In contrast, most of the humoral-immune genes showed strong down-regulation at 28 dpi (**Figures 6A, C–E**), only the *ighmc* was significantly upregulated (**Figure 6F**). This observation at 28 dpi is controversial but in line with the triggered humoral response against *R. salmoninarum* in salmonids, which does not necessarily correlate with immune protection (7, 8, 106).

*R. salmoninarum* persisted in the lumpfish tissues for at least 98 dpi (**Figure 2C**), which correlates with the chronic nature of BKD (2, 107). Significant upregulation of the eicosanoid *cox2* at 98 dpi (**Figure 5F**) could be related to the inflammatory response and supports the chronic persistence of *R. salmoninarum* in lumpfish tissues. *tnfα* was significantly upregulated at 98 dpi, which could be the result of MSA accumulation in infected lumpfish (32). Chronic stimulation of *tnfα* is known to assist the chronic inflammatory pathology of BKD and contributed to the host-mediated destruction of the kidney tissues in rainbow trout (32). In contrast, survivor lumpfish with considerable *R. salmoninarum* burden remaining in their internal tissues for at least 98 dpi (**Figure 2C**) did so in the absence of BKD clinical signs (**Figures 3E, J, O**) even with high expression of *tnfα* with respect to the control (**Figure 4K**). This immune pattern might be related to the chronic stage of *R. salmoninarum* infection. On the other hand, *tnfα* upregulation at 98 dpi (**Figure 4K**) could be linked to the low bacterial loads in lumpfish tissues at 98 dpi compared to 28 dpi (**Figure 2C**), because of the role of TNF-α in restricting the bacterial growth in infected macrophages and promoting macrophage survival in zebrafish (*Danio rerio*) infected *Mycobacterium marinum* (108).

Most of the downregulated adaptive immune genes (*igha, igh*δ, *ighma*, *ighmb, cd4a*, *cd4b*, *ly6g6f*, and *cd8a*) in infected lumpfish at 28 dpi returned to basal expression levels at 98 dpi (**Figures 6A, C–E, G–J**). Upregulation of *cd74* at 98 dpi (**Figure 6K**) could induce MHC-II expression. Also, significant stimulation of *ifnγ* at 98 dpi (**Figure 4J**) could enhance antigen presentation through MHC-I, as was observed in rainbow trout (109). Thus, the interaction between this intracellular pathogen and teleost MHC-pathways warrants further investigation.

Based on gene expression results, *R. salmoninarum* could immune-suppressed lumpfish at the early infection stages (28 dpi). In contrast, at late stages (98 dpi), it seems that *R. salmoninarum* is partially controlled by the lumpfish immune system, which may be attributed to the induced cell-mediated immunity. It is not clear whether the *R. salmoninarum* will be cleared or if it will persist and be horizontally transmitted or vertically transferred to the next generation of lumpfish. On the other hand, the majority of the lumpfish (65%) survived *R. salmoninarum* infection and presented the bacteria in head kidney until 98 dpi. These observations suggest that lumpfish is susceptible to *R. salmoninarum*. Lumpfish susceptibility to high virulent strains of *R. salmoninarum* with multiple *msa* gene copies (i.e*.*, *msa* gene copies ranged from two to five among 68 isolates) (110) and its transmission potential to other fish species warrants future research.

## Conclusion

This study revealed that lumpfish is susceptible to *R. salmoninarum* ATCC 33209 i.p infection, exhibiting a chronic infection pattern. *R. salmoninarum* caused immune suppression and modulated the lumpfish immune response towards the MHC-I pathway at 28 dpi. Lumpfish seemed to trigger a cell-mediated immune response against *R. salmoninarum* at the chronic stage of infection. Although *R. salmoninarum* persisted for at least 98 dpi in lumpfish tissues, it is not known whether lumpfish is able to clear the infection or if *R. salmoninarum* will persist and use lumpfish as a vector during cohabitation with salmon. Lumpfish susceptibility to more virulent *R. salmoninarum* strains or different routes of infection warrants further investigation.

## Data Availability Statement

The datasets presented in this study can be found in online repositories. The names of the repository/repositories and accession number(s) can be found below: https://www.ncbi.nlm.nih.gov/, SRP238224 and **Supplementary Material** (**Supplementary File S1**).

## Ethics Statement

All animal protocols required for this research were reviewed and approved by the Institutional Animal Care Committee and the Biosafety Committee at Memorial University of Newfoundland (MUN) (https://www.mun.ca/research/about/acs/acc/) based on the guidelines of the Canadian Council on Animal Care (https://ccac.ca/). Experiments were conducted under protocols #18-01-JS, #18-03-JS, and biohazard license L-01.

## Author Contributions

Conceptualization: JS and HG. Methodology: JS, HG, TC, AH, MD, JH, SK, DC, and DB. Investigation: HG, TC, MD, AH, JH, SK, DC, DB, and JS. Writing original-draft: HG, JH, and JS. Resources: JS and DB. Writing - Review & Editing: HG, TC, AH, MD, JH, SK, DC, DB, and JS. Visualization: HG and JS. Supervision: JS. Funding acquisition: JS and DB. All authors contributed to the article and approved the submitted version.

## Funding

This work was funded through grants from the Canada First - Ocean Frontier Institute to JS (sub-module J3), the Vietnam International Education Development (VIED) fellowship, and NSERC-Discovery grant (RGPIN-2018-05942).

## Conflict of Interest

The authors declare that the research was conducted in the absence of any commercial or financial relationships that could be construed as a potential conflict of interest.

## Publisher’s Note

All claims expressed in this article are solely those of the authors and do not necessarily represent those of their affiliated organizations, or those of the publisher, the editors and the reviewers. Any product that may be evaluated in this article, or claim that may be made by its manufacturer, is not guaranteed or endorsed by the publisher.
