# Supplementary material for: Lumpfish (Cyclopterus lumpus) Is Susceptible to Renibacterium salmoninarum Infection and Induces Cell-Mediated Immunity in the Chronic Stage
Source: Front Immunol. 2021 Nov 22;12:733266. doi: 10.3389/fimmu.2021.733266 (PMC8645940; doi:10.3389/fimmu.2021.733266)
Supplement: Supplementary file 1 [file DataSheet_1.zip › Supplementary Figure S5.pdf]

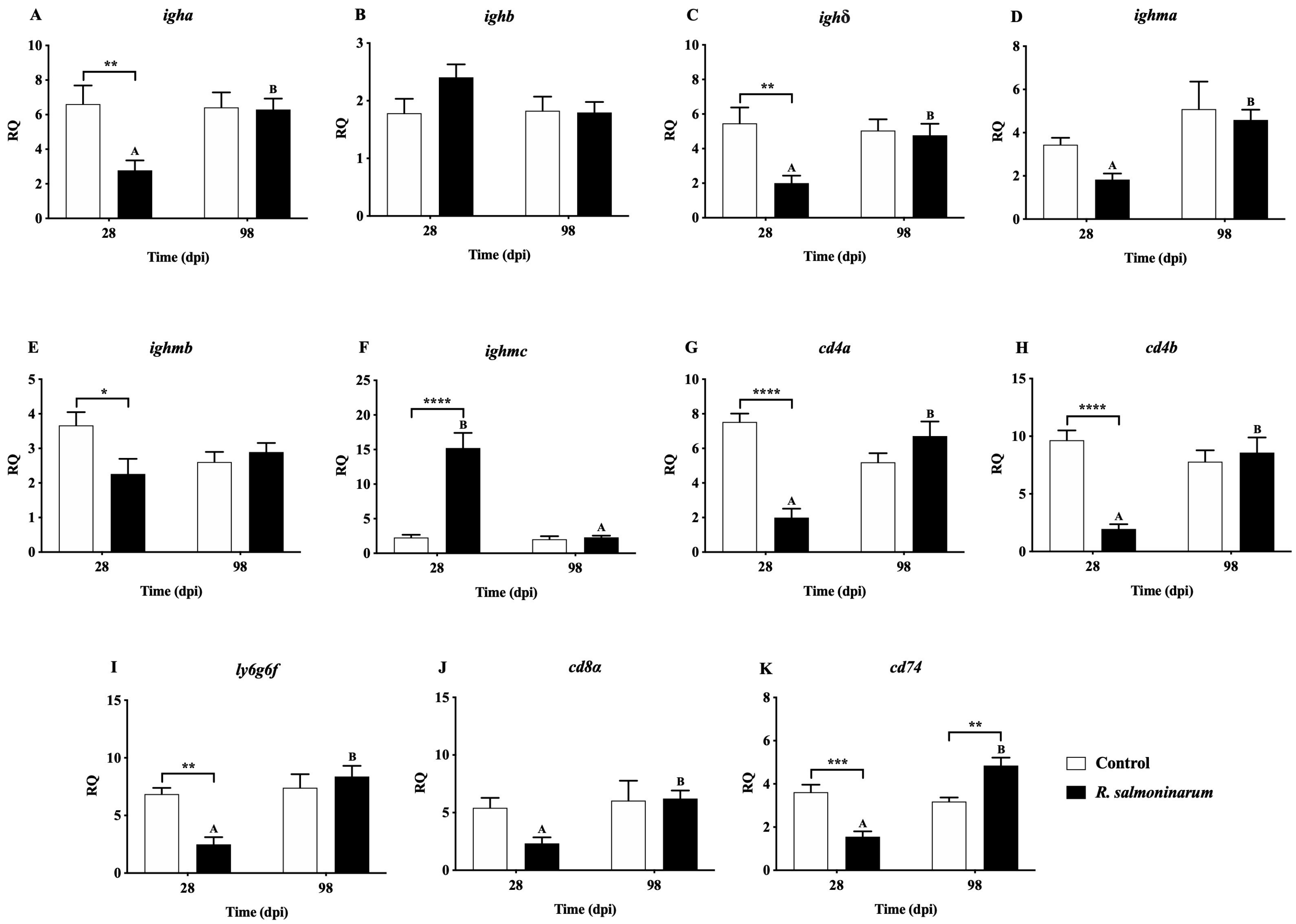

Supplementary Figure 5. Expression of transcripts related to humoral (A-F) and cellular mediated (G-K) immunity in lumpfish head kidney in response to *R. salmoninarum* infection at 28 and 98 days post infection (dpi). Transcript expression levels in head kidney from control (PBS-mock infected group) and infected [high dose (1×10^9 cells/dose) of *R. salmoninarum*] lumpfish at 28 and 98 dpi were analyzed using qPCR. Transcript levels are presented as relative quantity (RQ) values (i.e. values for the transcript of interest were normalized to both *etif3d* and *pabpc1b* transcript levels and were calibrated to the individual with the lowest normalized expression level of that given transcript). A two-way ANOVA test, followed by the Sidak multiple comparisons post hoc test was used to identify significant differences between treatments (control and infected groups) at a single time point, and for a given treatment at different time points (28 and 98 dpi). Asterisks (*) represent significant differences between treatments at each time-point (*$p < 0.05$, **$p < 0.01$, ***$p < 0.001$, ****$p < 0.0001$). Different letters represent significant differences between control (lower case) and infected (upper case) groups at 28 compared to 98 dpi. Each value is the mean ± S.E.M ($n = 6$).
