# Supplementary material for: Lumpfish (Cyclopterus lumpus) Is Susceptible to Renibacterium salmoninarum Infection and Induces Cell-Mediated Immunity in the Chronic Stage
Source: Front Immunol. 2021 Nov 22;12:733266. doi: 10.3389/fimmu.2021.733266 (PMC8645940; doi:10.3389/fimmu.2021.733266)
Supplement: Supplementary file 1 [file DataSheet_1.zip › Supplementary Figure S2.pdf]

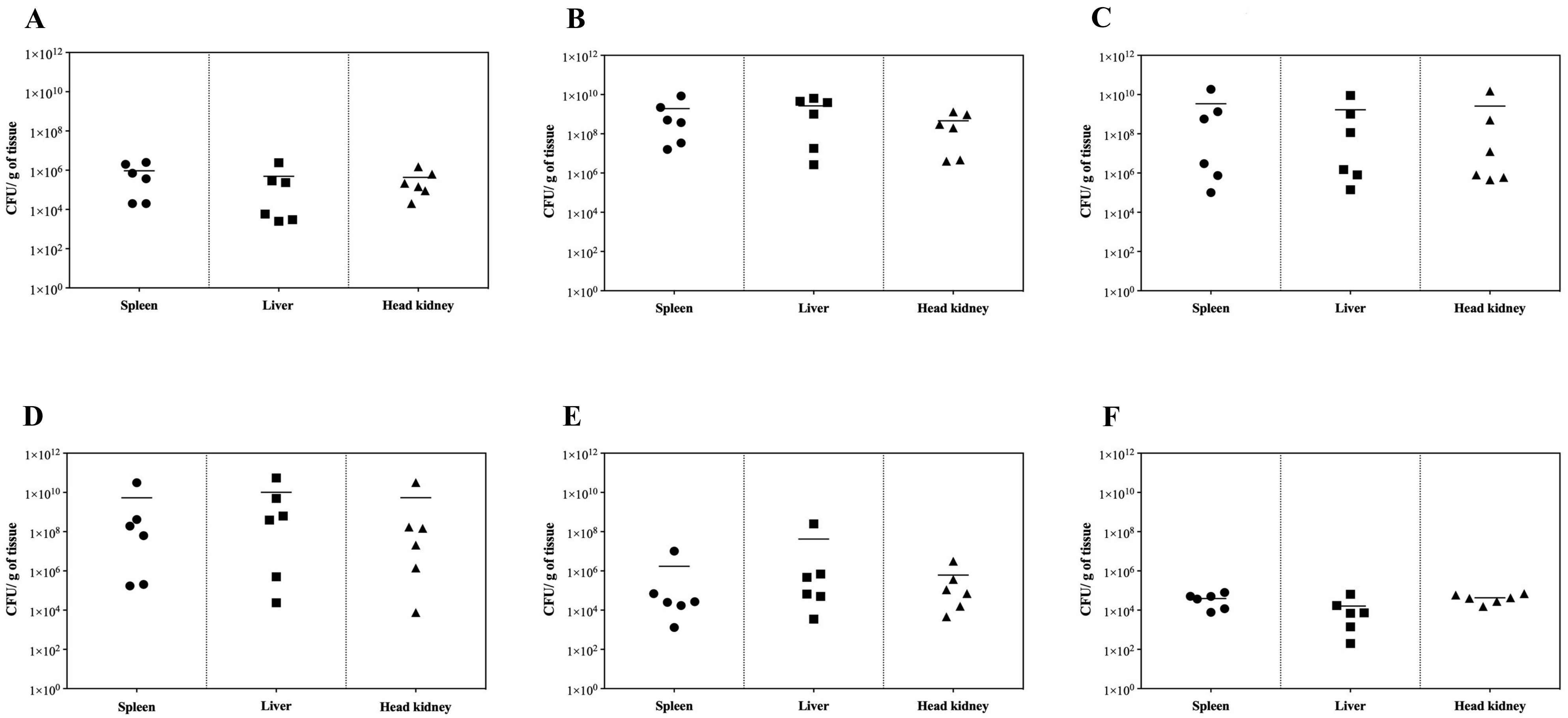

Supplementary Figure 2. *R. salmoninarum* colonization in high-dose infected lumpfish ($n = 6$) spleen, liver and head kidney at (**A)** 14, (**B)** 28, (**C)** 42, (**D)** 56, (**E)** 84 and (**F)** 98 days post infection. There were no significant differences in bacterial loads between the 3 tissues at each time point ($p<0.05$), but differences between individual fishes were significant ($p<0.05$) at all the time points except at 84 and 98 dpi, as determined by a one-way ANOVA test followed by the Holm-Sidak post hoc test to compare the differences between tissues, and within fish individuals, at a single time point.
