# Supplementary material for: Lumpfish (Cyclopterus lumpus) Is Susceptible to Renibacterium salmoninarum Infection and Induces Cell-Mediated Immunity in the Chronic Stage
Source: Front Immunol. 2021 Nov 22;12:733266. doi: 10.3389/fimmu.2021.733266 (PMC8645940; doi:10.3389/fimmu.2021.733266)
Supplement: Supplementary file 1 [file DataSheet_1.zip › Supplementary Figure S1.pdf]

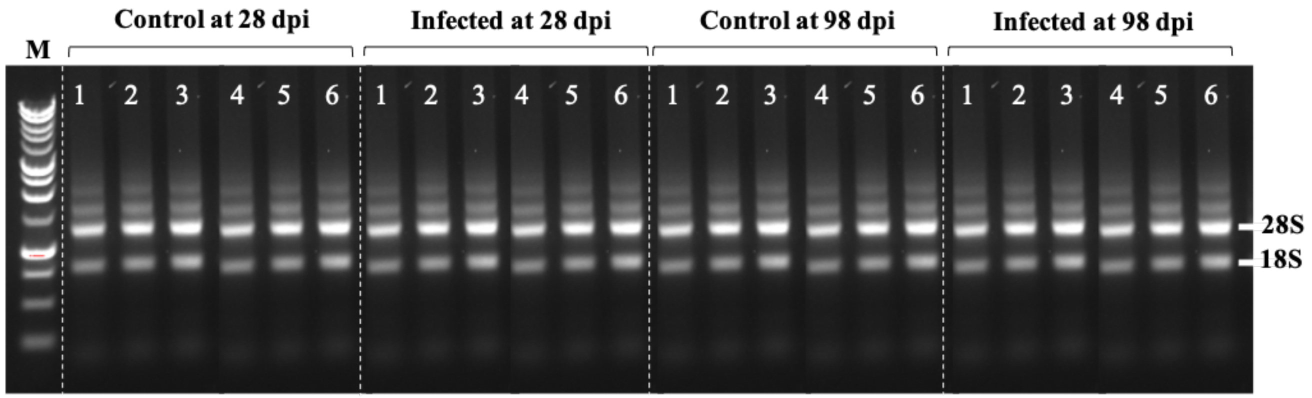

Supplementary Figure 1. Integrity of RNA samples extracted from the head kidney of control and high dose *R. salmoninarum* infected lumpfish at 28 and 98 days-post infection (dpi). Total RNA from the head kidney of control lumpfish at 28 dpi (n = 6), high dose *R. salmoninarum* infected lumpfish at 28 dpi (n = 6), control lumpfish at 98 dpi (n = 6) and high dose *R. salmoninarum* infected lumpfish at 98 dpi (n = 6) was extracted using TRIzol reagent (Invitrogen), and then purified using the RNeasy MinElute Cleanup kit (QIAGEN) following the manufacturers' instructions. RNA samples were treated with 2U of Turbo DNase (TURBO DNA-free™ Kit, Invitrogen) following the manufacturers' instructions. 1 µg of RNA was run on a 1% agarose gel with ethidium bromide staining. Crisp 28S and 18S ribosomal RNA bands at a 2:1 ratio were indicative of acceptable RNA integrity. M = 1 kb molecular weight marker (Promega, Fisher Scientific, CA).
